# LMP1 Up-regulates Calreticulin to Induce Epithelial-mesenchymal Transition via TGF-β/Smad3/NRP1 Pathway in Nasopharyngeal Carcinoma Cells

**DOI:** 10.7150/jca.37415

**Published:** 2020-01-01

**Authors:** Dongmei Ye, Junhui Zhu, Qiang Zhao, Wei Ma, Yiyang Xiao, Gaosheng Xu, Zhiwei Zhang

**Affiliations:** 1 Cancer Research Institute of Hengyang Medical College, University of South China; Key Laboratory of Cancer Cellular and Molecular Pathology in Hunan Province, Hunan Hengyang 421001, China.; 2 Department of Surgery, Innovative Practice Base for Postgraduate Training of Basic Medicine and Clinical Collaboration, University of South China and Yueyang Maternal and Child Health Hospital, Yueyang 414000, Hunan Province, China.; 3 Department of Pathology, The First Affiliated Hospital of University of South China, Hunan Hengyang 421001, Hunan Province China.; 4 Clinical Medicine of Hengyang Medical College, University of South China, Hengyang 421001, Hunan Province, China.

**Keywords:** nasopharyngeal carcinoma, latent membrane protein 1, calreticulin, Epithelial-mesenchymal transition, TGF-β signaling pathway, Neuropilin-1

## Abstract

**Background**: Latent membrane protein 1 (LMP1) is known as an oncogenic protein encoded by the EBV genome. The purpose of this study was to investigate the mechanism of LMP1-induced cell epithelial-mesenchymal transition (EMT).

**Methods**: The NP69 cell line of nasopharyngeal epithelial cells with high expression of LMP1 was established to observe the effect of high expression of LMP1 on cell growth, proliferation, cycle, apoptosis, migration and invasion. We used proteomics to screen and identify differentially expressed proteins related to LMP1-mediated epithelial cell transformation. Then, we analyzed the expression and significance of differentially expressed calreticulin (CRT) in nasopharyngeal carcinoma (NPC), and observed the effect of CRT expression on EMT in CNE2 cells of NPC. Finally, the expression of neuropilin-1 (NRP1), which is a protein downstream of the EMT-related signaling pathway TGF-β (transforming growth factor β), was detected.

**Results**: LMP1 promoted NP69 cells proliferation, inhibited apoptosis and induced EMT. We identified 22 differentially expressed proteins associated with LMP1-induced EMT. Among them, CRT expression level was significantly increased in NPC compared with adjacent tissues, and was interrelated with TNM staging and lymph node metastasis of NPC. After knockdown of CRT expression, the phenomenon of cell EMT was reduced and the ability of cell migration and invasion was weakened. CRT regulated NRP1 expression by affecting SMAD3 phosphorylation.

**Conclusion**: LMP1 induced cell EMT via TGF-β/Smad3/NRP1 pathway, which promoted migration and invasion of NPC cells.

## Introduction

Nasopharyngeal carcinoma (NPC) is a common malignant tumor in Southeast Asia. Recurrence and metastasis are the main causes of death in patients with NPC^1^. The occurrence of NPC is associated with Epstein-Barr virus (EBV) infection, and EBV-encoded Latent membrane protein 1 (LMP1) promotes proliferation, migration and invasion of NPC cells^2^-^5^. LMP1 is known to be a vital oncogenic protein encoded by the EBV genome, as it plays a critical role in the transformation of rodent fibroblasts and some immortalized epithelial cells in vitro^6^-^7^.

LMP1 is a 60-66 kD, fully phosphorylated transmembrane glycoprotein consisting of 386 amino acid (aa) residues, including hydrophilic amino-terminal cytoplasmic region (1-23aa), six different hydrophobic transmembrane regions (24-186aa), and a hydrophilic carboxyl-terminal cytoplasmic region (187-386aa). The carboxy-terminal cytoplasmic domain is the activation of receptor transduction intracellular signal transduction, which is the main site of tumorigenesis, including three carboxy terminal activating regions (CTAR), namely CTAR1, CTAR2 and CTAR3^6^. LMP1 involves multiple functional activation regions and complex signaling pathways that activate its downstream signaling pathways^7^, such as IKKα/IKKβNF-B, SEK/JNK/C-JUN/AP-1 and JAK3/STAT.

Subsequently, we screened and identified the protein-CRT closely related to EMT from the immortalized nasopharyngeal epithelial cells NP69 stably expressing LMP1 by proteomics. CRT is widely present in eukaryotic cells and is a highly conserved multifunctional endoplasmic reticulum protein^8^. CRT is overexpressed in a variety of malignant tumors including pancreatic cancer, lung cancer, and breast cancer, leading to a poor prognosis^9^-^11^. Shi et al. have indicated that the CRT-STAT5A-NRP1 pathway promotes migration and invasion of esophageal squamous carcinoma cells, and NRP1 expression is positively correlated with CRT expression in esophageal squamous cell carcinoma, suggesting that this pathway-related gene is a potential molecular therapeutic target^12^. In addition, NRP1 promotes metastasis of NPC by up-regulating invasive-associated proteins to induce EMT^13^-^14^. The subject of this study was to investigate the mechanism and signaling pathway of LMP1 up-regulating CRT-induced EMT in NPC cells. Morever, it provides a new experimental and theoretical basis for elucidating the molecular mechanism of invasion and metastasis of NPC.

## Materials and Methods

### Cell line and cell culture

The retroviral vector pLNSX and pLNSX-LMP1^WT^ (wild type) were kindly provided by Dr Liang Cao (University of Hong Kong, Hong Kong SAR, China). We have previously established the NP69-pLNSX and NP69-LMP1^WT^ cell lines^15^. NPC CNE2 cells and SV40-immortalized nasopharyngeal epithelial cells NP69 were received from Cancer Research Institute of Central South University and Dr Sai Wah Tsao (University of Hong Kong), respectively. The cells were cultured in RPMI-1640 (HyClone) containing 10% fetal calf serum (Hangzhou, Sijiqing, China) or serum-free keratinocyte medium (K-SFM; Gibco; Thermo Fisher Scientific, Inc., Waltham, MA, USA) in humidified 5% (v/v) CO2 atmosphere at 37 °C.

### Patients

We collected 52 NPCs and 57 non-carcinomas tissues that were diagnosed in the Department of Pathology of the First Affiliated Hospital of University of Southern China. All samples were obtained from patients of NPC with approval of the medical ethics committee.

### Immunofluorescence assay

Cell slides were prepared, and incubated with mouse anti-human monoclonal antibodies S12 for LMP1 (1:50, a generous gift from Dr Liang Cao of University of Hong Kong) for 1 hour at 37 °C. After washing, the FITC-labeled goat anti-mouse secondary antibody (1:500, Zymed, USA) was marked, and then the cells were observed by fluorescence microscope (EVOS FLAuto, Life technologies, USA).

### Growth curve assay

NP69-pLNSX and NP69-LMP1^WT^ cells were inoculated into 96-well plates in triplicate at 1×10^4^ cells/well. The number of cells was detected by MTT method according to the manufacturer's instructions (Molecular Probes, Eugene, OR, USA) every 48 hours. The OD values were measured at 570 nm by a microplate reader (17260, BIO-RAD, USA).

### Colony formation assay

The cells were made into a cell suspension, and seeded in a six-well plate at 1×10^3^ cells/well at 37℃ for 2 weeks. The colonies were fixed with methanol for 15min and then stained with 0.4% crystal violet (C0121, Beyotime Biotechnology; Shanghai, China). The number of cell colonies was counted by the microscope (TS100, Nikon, Japan) and analyzed.

### Soft agar clone formation assay

Cells were seeded into semisolid agar K-SFM medium [base layer, 0.6% (w/v); upper layer, 0.3% (w/v)] at a density of 5×10^4^ cells/well in 6-well plates. After 2 weeks of incubation at 37 °C with 5% (v/v) CO2, the number and the size of colonies (≥50 cells as one colony) were observed and analyzed.

### Flow cytometry

The effect of LMP1^WT^ on cell-cycle distribution and apoptosis of NP69 cells was detected using flow cytometry. NP69-pLNSX and NP69-LMP1^WT^ cells were separately collected and then centrifuged at 2000 rpm for 5 min. After fixing with pre-cooled 70% ethanol for 24 hours at 4 °C, the distribution and apoptosis of each phase of the cell cycle were measured by flow cytometry (Becton Dickson Immunocytometry Systems, San Jose, CA, USA) for 1×10^6^/L cells.

### Hoechst33258 staining

The cells were seeded on coverslips, and then 1.0 mg/L of paclitaxel was added and cultured for 24 hours. Cells were fixed with methanol, and Hoechst33258 solution was added according to the instructions. After incubation at 37 °C for 10 minutes, it was observed the nuclei of independent cells condensation with a fluorescence microscope (EVOS FLAuto, Life technologies, USA) and imaged.

### MALDI-TOF MS and database analysis

Total cellular protein was extracted using lysis buffer (7 mol/l urea, 2mol/l thiourea, 2% (v/v) NP-40, 1% (v/v) Triton X-100, 100 mmol/l dithiothreitol (DTT), 5 mmol/l PMSF, 4% (w/v) CHAPS, 0.5 mmol/l EDTA, 40 mmol/l Tris, 1 mg/ml DNase I). The concentration of the total proteins was assayed with 2D Quantification kit (GE Healthcare). Differentially expressed proteins were separated by two-dimensional (2D) gels electrophoresis. We analyzed protein spots with Applied Biosystems Voyager System 4307 MALDI-TOF mass spectrometer (Applied Biosystems; Thermo Fisher Scientific, Inc.). The trypsin fragment peak was used as the internal standard for quality calibration. A list of the corrected mass peaks was the peptide mass fingerprinting (PMF). Proteins were identified with PMF data via searching (Matrix Science, London, UK). In addition, we established a protein signaling network for LMP1 promote EMT in SV40-immortalized nasopharyngeal epithelial cell line NP69 through bioinformatics software (http://david.abcc.ncifcrf.go).

### Immunohistochemical staining

Samples were fixed with 10% formalin and 4um paraffin sections were baked in a 60 °C incubator for 2 hours. The immunohistochemical staining procedure was performed following the S-P kit instructions (Maixin, Fujian, China). It was Incubated with Calreticulin antibody (1:500, ab92516, Abcam, UK), LMP1 (1:1000, ab78113, Abcam, UK), overnight at 4 °C, and stained by adding DAB. Finally, the cells were observed with microscope (BX53, Olympus, Japan).

### Transfection

Transfections were performed with the riboFECTTMCP Reagent (Ruibo, Guangzhon, China) according to the manufacturer's protocol. Cells were seeded in a 6-well plate at 1×10^5^/well, transfected with siRNA (target sequence: GCACCAAGAAGGTTCATGT, synthesized by Ruibo, Guangzhou, China), and cultured for 24 hours. Transfection efficiency was observed by a fluorescence microscope.

### Western-blot analysis

Total cellular proteins were separated via 8% SDS-PAGE gels and transferred to polyvinylidene difluoride membranes. The membranes were blocked with 5% fat-free milk for 1 hour, incubated with antibodies Calreticulin (ab92516, 1:500, Abcam, UK), Vimentin (5741, 1:1000, CST, USA), SMAD3 (9523T, 1:1000, CST, USA), P-SMAD (C25A9, 1:1000, CST, USA), NRP1 (abs120133, 1:250, Aibixin, Shanghai, China), E-Cadherin (3195, 1:500, CST, USA), MMP9 ( ab137867, 1:1000, Abcam, UK), TGF-β (ab9248, 1:250, Abcam, UK), β-actin (1:1500; Sigma-Aldrich; Merck KGaA, Darmstadt, Germany) at 4 °C overnight, and fluorescent secondary antibody (1:2000; Santa Cruz Biotechnology, Inc.) for 2 hours. The blots were detected by the Gel imaging analysis system (ODYSSEY Sa, LI-COR, USA).

### Wound healing assay

The cell suspension were inoculated into 1×10^5^ cells per well in 6-well plates. Cells were transfected with Si-CRT and treated with 10 µM SIS3 (Specific inhibitor of SMAD3), respectively, when the cell density was approximately 80%. We used untreated cells as a control group. Then, we scratch the cell monolayer with a 10ul pipette tip. The culture was continued in a medium containing 3% fetal bovine serum at 37 °C with 5% CO2 incubator. Photomicrographs at 1×100 magnification were captured at 0 and 36 hours after scratches were made by computer-assisted microscope (EVOS FLAuto, Life technologies, USA).

### Transwell migration and invasion assay

The transwell invasion assay was performed with the transwell (Corning, USA) and matrigel (GIBCO, USA) following the manufacturer's instructions. Cells at a density of 2×10^5^ per well were inoculated into the upper layer of the chamber, and add the medium containing 10% fetal bovine serum to the lower layer, incubated for 24 hours at 37 °C with 5% CO2. The Matrigel gel was wiped off and fixed with 4% polymethanol for 30 min. The number of cells was counted by staining with crystal violet. The steps of the transwell migration assay were the same as the invasion assay, except that Matrigel gel was not placed at the bottom of the chamber.

### NRP1 promoter analysis

We searched the 2000bp sequence of bases promoter sequence from the Ensembl site (http://asia.ensembl.org/index.html) (Table 1). We Searched and analyzed potential transcription factor binding sites via the Jaspar website (http://mbs.cbrc.jp/research/db/TFSEARCH.html).

### Statistical Analysis

Statistical analyses were performed using SPSS 20.0 software (IBM Corp, Armonk, NY, USA). Data were expressed as mean±standard deviation (M±SD). One-way ANOVA was used to compare the mean of samples between groups. Student's t-test was performed when the variances between groups are similar, and the correlation test was used by Spearman's correlation analysis. *P<0.05, **P<0.01, ***P<0.001 were considered to indicate a statistically significant difference.

## Results

### LMP1^WT^ expression affects biological behavior of nasopharyngeal epithelial cells NP69

**Figure 1:**
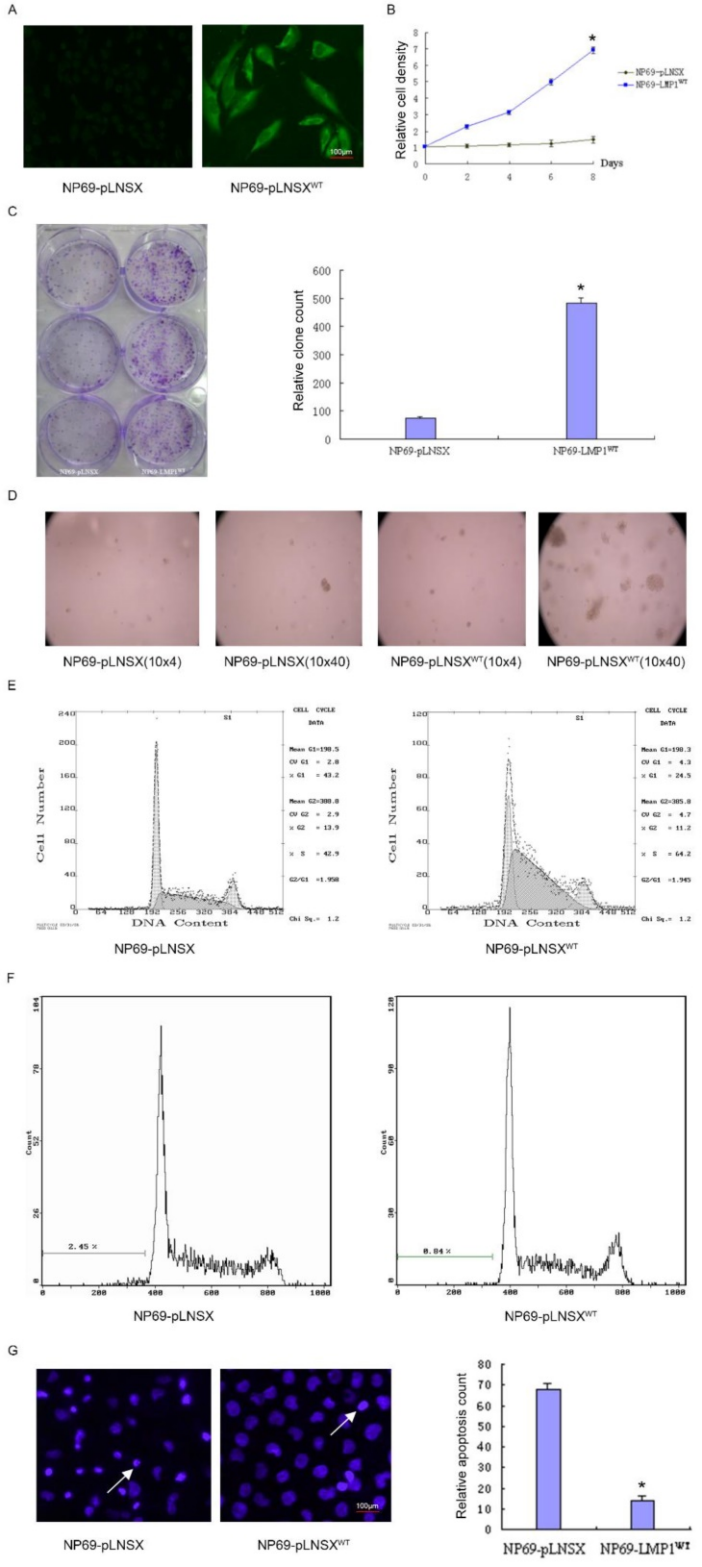
LMP1^WT^ Expression affects biological behavior of nasopharyngeal epithelial NP69 cells. **(A)** Immunofluorescence assays were performed to observe the LMP1^WT^ which was mainly localized within the cell membrane and cytoplasm in NP69 cells (1×40). **(B)** The NP69-LMP1^WT^ cells growed faster than NP69-pLNSX cells **(C)** Colony forming ability of NP69-LMP1WT cells was significantly increased compared with NP69-pLNSX cells. **(D)** The NP69-LMP1^WT^ cells soft agar clones were larger, closely packed and round in shape. **(E-F)** Cell cycle distribution and apoptosis cells of NP69-pLNSX and NP69-LMP1^WT^ were assessed by flow cytometry. **(G)** Apoptosis of NP69-LMP1^WT^ cells was decreased by hoechst33258 staining, as indicated by the arrow. P-values are presented as the mean ± standard deviation (n=2, *P<0.05 vs. NP69-pLNSX). LMP1, latent membrane protein 1; WT, wild type. Data are shown as mean±SD. *P<0.05.

To investigate the potential role of LMP1^WT^ in NP69 cells, we have previously established the NP69-LMP1^WT^ cell line^15^. By immunofluorescence staining, it was observed that LMP1^WT^ protein was mainly localized within the cell membrane and cytoplasm (Figure 1A). Subsequent growth curve assays indicated that high expression of LMP1^WT^ significantly promoted the growth of NP69 cells (Figure 1B). In colony formation assays and soft agar clone formation assays, colony forming ability of NP69-LMP1^WT^ cells was significantly increased compared with NP69-pLNSX cells (Figure 1C-D). Then, we evaluated the effect of LMP1^WT^ on NP69 cell cycle and cell apoptosis by flow cytometry assays. The results showed that NP69-LMP1^WT^ cells in G0 and G1 phase were significantly reduced compared with NP69-pLNSX cells, while cells in S phase were significantly increased (Figure 1E-F, Table 1). Apoptosis was detected by hoechst33258 staining. We found that LMP1^WT^ suppressed apoptosis in NP69 cells (Figure 1G, Table 2). Our results showed that LMP1^WT^ was involved in the regulation of cell cycle, promoting growth and proliferation, inhibiting apoptosis in NP69 cells.

### Identification of LMP1-induced cell EMT related protein

**Figure 2:**
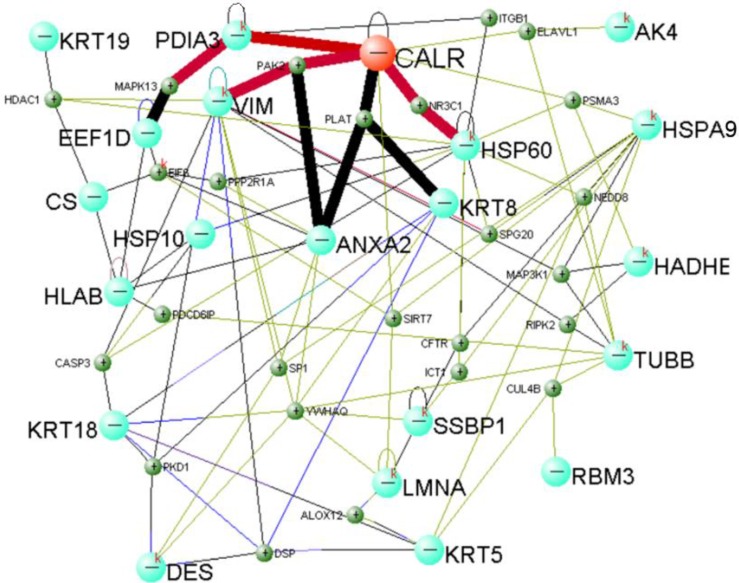
Calreticulin-mediated LMP1 signaling network that promotes EMT in NP69 cells.

Total protein of NP69-pLNSX and NP69-LMP1 cells was separated from two-dimensional gel electrophoresis (2-DE). We used matrix-assisted laser desorption/ionization time-of-flight mass spectrometry (MALDI-TOF-MS) to screen and identify 22 differentially proteins expression from LMP1-induced EMT including Calreticulin (Table 3). In addition, previous Fluorescent real-time quantitative RT-PCR and Western blot analysis were consistent with proteomics results^15^. We established Calreticulin-mediated LMP1 promotes the signaling pathway of EMT in NP69 cells via DAVID Online Bioinformatics Analysis Software (http://david.abcc.ncifcrf.gov) (Figure 2). Among them, CALR-PDIA3-MAPK13, CALR-PAK2-VIM and CALR-NR3C1-HSP60 are three important interaction pathways.

### Expression and clinical significance of calreticulin in NPC

**Figure 3:**
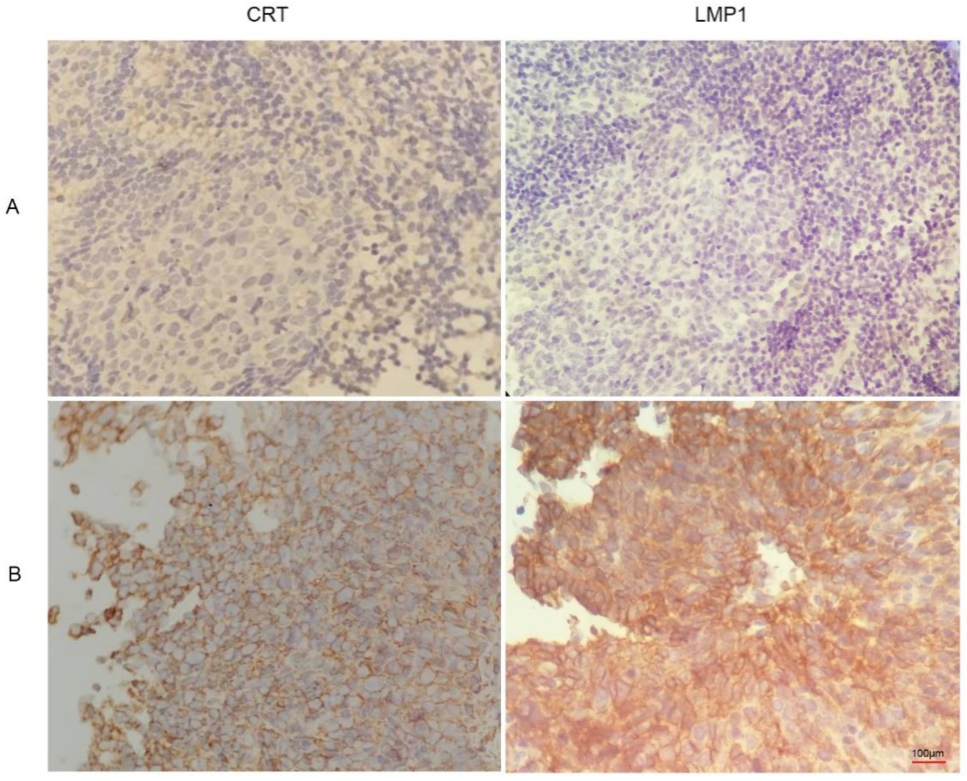
Immunohistochemistry images of CRT and LMP1 in NPC and nasopharyngeal inflammatory epithelium (4x100). **(A)** nasopharyngeal inflammatory tissue. **(B)** NPC tissue.

We found that CRT was highly expressed in NPC tissues with LMP1 positive expression (Figure 3). Subsequently, we evaluated the expression and clinical significance of CRT in NPC. Immunohistochemical (IHC) staining analysis revealed that CRT was highly expressed in 82.69% (43/52) NPC tissues, whereas 19.29% (11/57) nasopharyngeal benign lesions expressed CRT (P<0.05) (Table 4). Subsequently, we explored the correlation with CRT expression and clinicopathological status of NPC patients. We found that in 52 NPC samples, CRT expression in NPC with clinical stage II-III and lymph node metastasis significantly increased compared with clinical stage I and no lymph node metastasis of NPC. According to statistical analysis, CRT expression was positively associated with clinical stage and infiltrated cervical lymph node (P=0.018). However, there were not associated with the patient's gender and age upon CRT expression (Table 5).

### Knockdown of CRT expression inhibits EMT, migration and invasion in NPC cells

**Figure 4:**
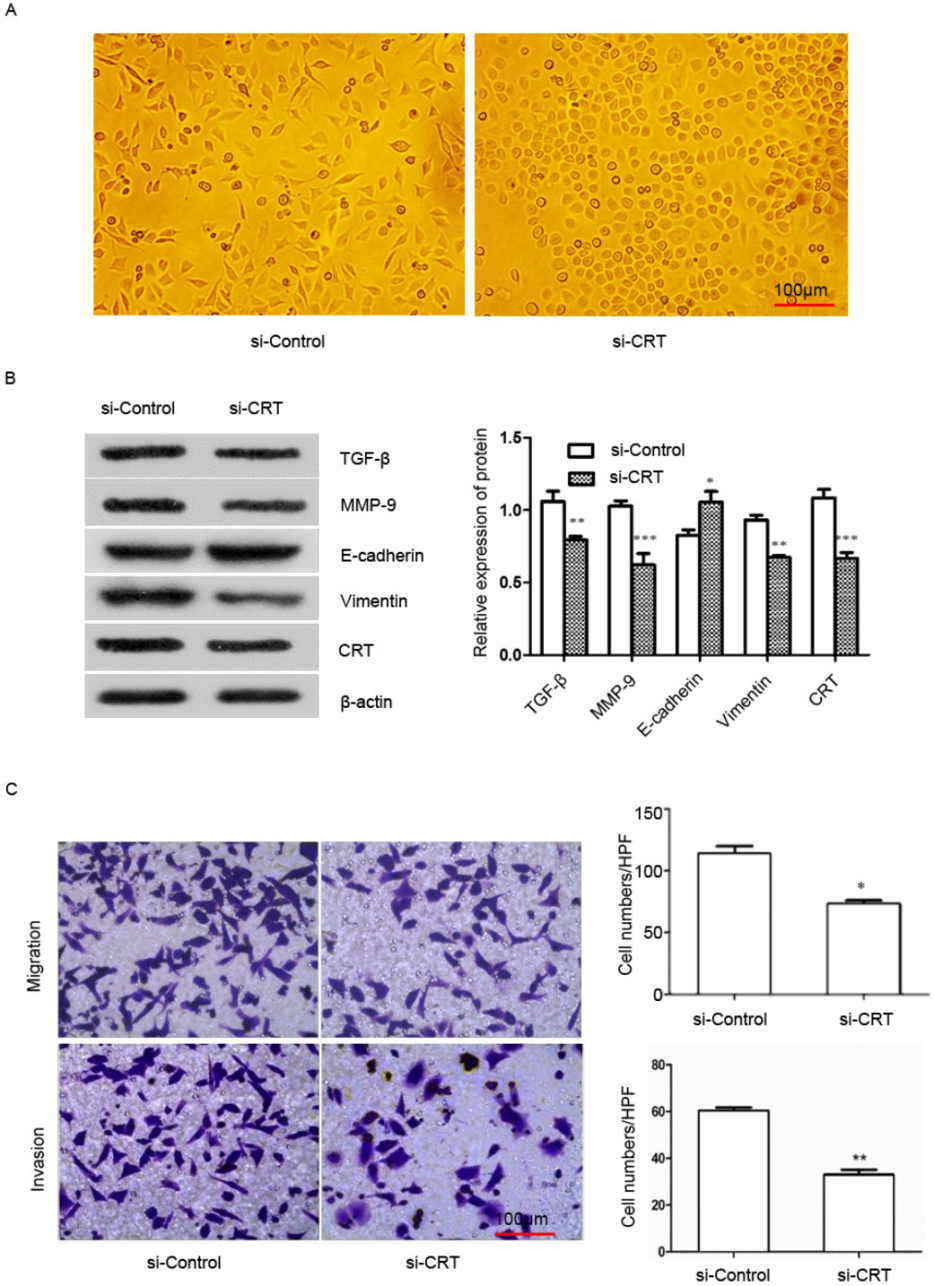
Silencing of CRT expression inhibits EMT, migration and invasion of NPC CNE2 cells. CNE2 cells were transfected with CRT-specific si-RNA (si-CRT) and si-Control, respectively. **(A)** Morphologies of si-CRT and si-Control NPC CNE2 cells. **(B)** The effect of Silencing of CRT expression on E-cadherin, vimentin, MMP-9 and TGF-β protein expression was measured by Western blot. **(C)** NPC CNE2 cell migration and invasion images and data analysis after Silencing of CRT (expression1x200). Data are shown as mean±SD. ^*^P<0.05, ^**^P<0.01, ^***^P<0.001.

To examine the effect of CRT expression on EMT migration and invasion of NPC cells, we transfected si-RNA (si-CRT) and si-Control into NPC CNE2 cells. Then we observed cell morphology and found that silencing CRT expression within cells induced a morphological change from a long fibroblastoid shape to an elliptical polygonal or cobblestone-like, with cells arranged closely (Figure 4A).

We used Western blot analysis to detect the expression of E-cadherin, vimentin, matrix metalloenzyme 9 (MMP-9) and transforming growth factor-β (TGF-β) in CNE2 cells. Our results showed that knockdown of CRT, the expression of E-cadherin was significantly up-regulated in si-CRT group compared with control group (si-Control), whereas the expression of vimentin, MMP-9 and TGF-β were significantly down-regulated (Figure 4B). Furthermore, we observed the effect of knocking down CRT expression on CNE2 cells invasion ability cells, in migration and invasion assays. As shown in Figure 4C, the migration and invasion ability of cells were markedly reduced in the si-CRT group.

### Bioinformatics predicted the transcriptional regulatory sites of NRP1

**Figure 5:**
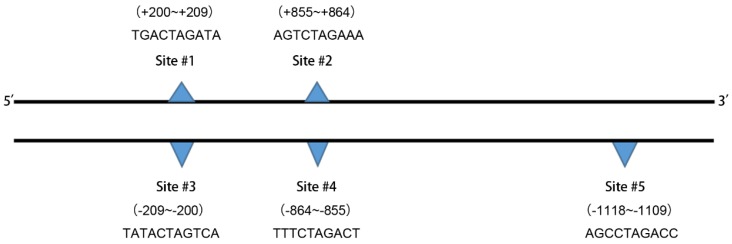
The binding site sequences are shown.

We used the Ensemble database (http://asia.ensembl.org/index.html) to search for a nucleotide sequence of 2,000 bases (-1~-2000) upstream of the transcription initiation site of the NRP1 gene (The promoter region is generally considered to be a DNA fragment of 1000bp upstream of the transcription start site), which is a promoter subsequence of NRP1 (Supplementary Table 1). The TGF-β signaling pathway is a classical signaling pathway that induces EMT. Morever, we used JASPAR (http://jaspar.genereg.net/) to explore whether the relevant transcription factors in TGF-β pathway protein family are involved in the regulation of NRP1 expression.We found that transcription factors with a Relative profile score threshold of 80% were shown in Table 6. There are five binding sites for transcription factor SMDA3 in the promoter region of NRP1 (Figure 5), the underlined part of Supplementary Table 1 is the two binding sites of SMAD3 on the NRP1 promoter gene sense strand.

### Regulation relationship CRT, SMAD3 and NRP1

**Figure 6:**
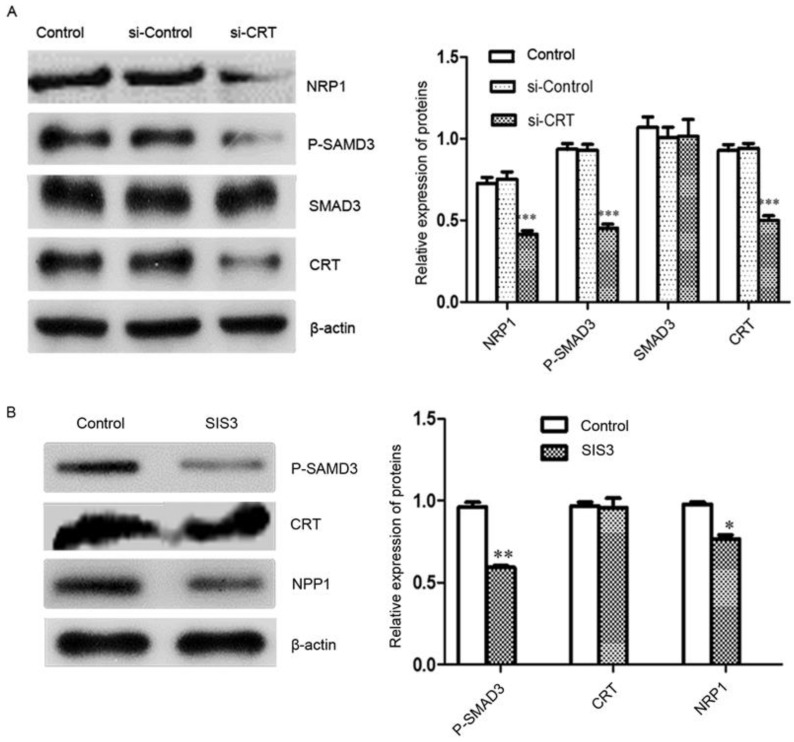
CRT regulates NRP1 expression by SMAD3. **(A)** Western blot was performed to detect the expression of CRT, NRP1, SMAD3 and P-SMAD3 in si-CRT group, non-specific siRNA (si-Control) and control group after knockdown of CRT expression. **(B)** CNE2 cells were treated with SIS3 at a concentration of 10 µM for 8 hours, and the untreated group was used as a control group. SIS3-treated CNE2 cells showed less NRP1 and P-SMAD3 expression compared with the control group by Western blot. Data are shown as mean±SD, ^*^P<0.05, ^**^P<0.01, ^***^P<0.001.

Signal transduction involves signal transmission and amplification from transmembrane receptors to the nucleus. Reversible phosphorylation of proteins is one of the main channels for regulating information. Phosphorylation plays a key role in the transmission of information in signaling pathways. Subsequently, we investigated the effect of knockdown of CRT expression on the transcription factor SMAD3 and its phosphorylation and NRP1 expression level in cells. We performed Western blot analysis, it revealed that Si-CRT did not change the expression levels of SMAD3, but illustrious reductions in P-SMAD3 protein level was found, Smad3 phosphorylation level was notablely inhibited and NRP1 expression was markedly reduced compared with Si-Contro and untransfected (control) (Figure 6A). To further confirm, we used inhibitor SIS3 to block TGF-β pathway by specifically inhibiting the phosphorylation of SMAD3. We found that P-SMAD3 was significantly down-regulated in the SIS3 inhibitor group, and NRP1 protein level was also significantly reduced compared with Si-Contro and control group. However, the SIS3 inhibitor did not chang the expression of CRT (Figure 6B).

### Effect of CRT/SMAD3 on migration and invasion of NPC cells

**Figure 7:**
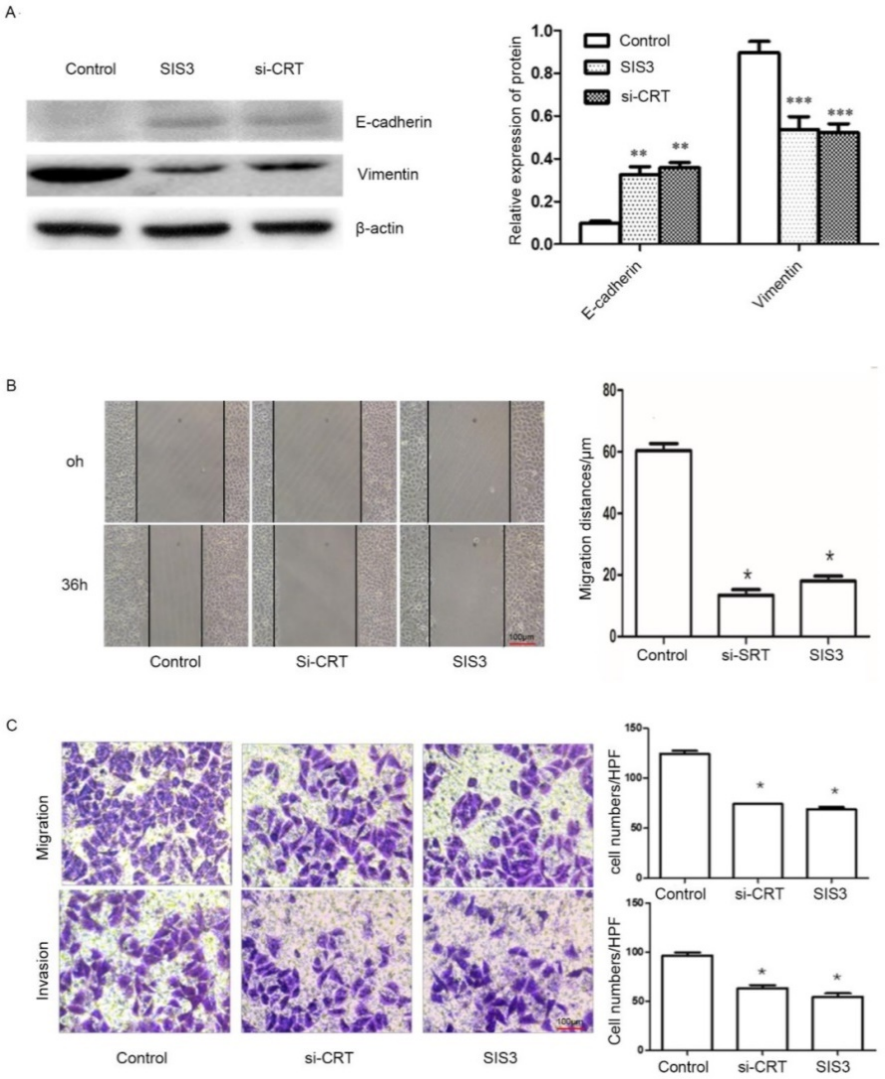
Effect of CRT/SMAD3 on EMT and migration in NPC CNE2 cells. **(A)** The effect of CNE2 cells treated with SIS3 inhibitors and transfected Si-CRT on Vimentin and E-cadherin protein expression was determined through Western blot analysis. **(B)** Wound healing assay of NPC CNE2 cells among control group, SIS3 inhibitors group and transfected with Si-CRT group (1x100). **(C)** Transwell migration and invasion assays of NPC CNE2 cells among control group, SIS3 inhibitors group and transfected with Si-CRT group (1x200). Data are shown as mean±SD. ^*^P<0.05, ^**^P<0.01, ^***^P<0.001.

In addition, we determined whether CRT/SMAD3 affects the ability of migration and invasion in NPC CNE2 cells. NPC CNE2 cells were treated with SIS3 and transfected with Si-CRT and cultured for 24 hours, respectively, and untreated as a control group. Then total cellular proteins were extracted. Western blot analysis was used to detect the expression of invasion-related proteins. The results revealed that SIS3 and Si-CRT groups significantly decreased expression levels of Vimentin, while noteblely increased the expression of E-cadherin compared with the control group (Figure 7A). Then, we analyzed the diversification of migration ability after treatment with SIS3 inhibitors and transfection of Si-CRT in cells through Wound healing assays. We found that in 0-36 hours, the migration ability of cells in Si-CRT group and SIS3 inhibitors treatment group was inhibited compared with the control group, while there was no significant difference of the migration distance between SIS3 inhibitors treatment groups and Si-CRT groups (Figure 7B). Further, we used migration and invasion assays to further confirm our results. We found that the number of cells migration and invasion in the Si-CRT groups and the SIS3 inhibitor groups was significantly decreased compared with the control group, wherea the counts of cell migration and invasion in the Si-CRT groups and the SIS3 groups were not significantly difference (Figure 7C).

## Discussion

Epstein-Barr virus is one of the earliest discovered human DNA tumor viruses, which is closely associated with various malignant tumors; however, its carcinogenic mechanism is still unclear. Almost all NPC occurrences are associated with EBV infection. Previous studies have revealed that EBV-encoded LMP1 was expressed in most NPC tissues and confirmed that LMP1 plays a vital role in the development of NPC^16^-^17^. However, there was no direct evidence that LMP1 induced NPC. We have previously found that NP69-LMP1^WT^ cells line stably expressing LMP1 gradually induced cells a morphologic change from elliptical polygonal or cobblestone-like to long fibroblastoid shape compared with the control cell NP69-pLNSX, and reduced intercellular contact^15^. Based on this study, we further confirmed that wild-type LMP1 induced cell proliferation and suppressed cell apoptosis by gain-of-function assays, similar to previous studies^18^-^19^. It was confirmed that LMP1 has a tumor gene function that promotes cell proliferation and transformation.

We used high-throughput proteomics to screen and identify the protein CRT, which was closely associated with EMT, from the NP69-LMP1^WT^ cell line^20^-^21^. We found that Real-time PCR and Western blot analysis confirmed the results of proteomics^15^. Subsequently, we established a signal network in which CRT mediates LMP1 to promote EMT in NP69 cells through bioinformatics analysis. Among them, CALR-PDIA3-MAPK13, CALR-PAK2-VIM and CALR-NR3C1-HSP60 are three vital interaction pathways.

CRT is an endoplasmic reticulum resident protein with a molecular weight of 46 kDa. Its initial function is to maintain intracellular calcium homeostasis and as a molecular chaperone. Studies have shown that CRT regulates biological processes such as cell cycle, proliferation and apoptosis via involving in various signal protein interactions in cells. CRT is up-regulated in a variety of tumors and is closely related to tumor progression, migration and invasion^20^-^22^. In our study, we demonstrated that the expression of CRT in NPC was significantly increased compared with adjacent non-cancerous tissues. Moreover, high expression of CRT was positively correlated with NPC clinical stage and neck lymph nodes infiltration as assessed via detecting CRT expression in 109 cases of NPC patients. After silencing the expression of CRT in CNE2 cells by specific siRNA, si-CRT induced cells a morphologic change from long fibroblastoid shape to elliptical polygonal or cobblestone-like, the cells were in intimate contact and inhibited cellular EMT. Our results provide evidence that CRT is a cancer-promoting molecule.

CRT mediates EMT by regulating multiple signaling pathways. The transforming growth factor β (TGF-β) signaling pathway is the classical pathway for the induction of EMT. Recent studies have shown that CRT can interact with TGF-β receptors I and II. Overexpression of CRT, TGF-β promotes Snail2/Slug inhibition of E-cadherin expression by mediating the inactivation of glycogen synthase kinase 3β^23^. Our current evidence revealed that CRT induced EMT by regulating TGF-β expression in NPC CNE2 cells.

Previous studies have shown that NRP1 is a downstream regulatory protein of CRT in esophageal squamous cell carcinoma^24^. As with previous studies, we found that NRP1 expression levels decreased with the down-regulation of CRT expression by Western blot analysis, indicating that NRP1 is a downstream regulatory protein of CRT in NPC cells. NRP1 is a non-tyrosine transmembrane glycoprotein that is highly expressed in various malignant tumors such as NPC^25^ and inhibits angiogenesis, cell proliferation and invasiveness of NPC^26^. Studies have demonstrated that TGF-βI activation, which is mediated by NRP1, is associated with regulatory T cell activity and tumor biological behavior^27^-^28^. TGF-β-induced EMT is mainly mediated by the SMAD pathway, and activated TβR-I can specifically recognize and bind to Smad2 and Smad3. Smad2/Smad3 plays an important role in the biological effects of TGF-β, as it is the first signaling molecule for TGF-β pathway-mediated activation^29^-^30^. There are five binding sites in the promoter region of NRP1 for transcription factor SMAD3 have been predicted using bioinformatics software. We also demonstrated that the expression level of NRP1 was significantly down-regulated following the inhibition of SMAD3 phosphorylation by pathway inhibition experiments. Our results indicated that CRT regulated NRP1 expression via the TGF-β/SMAD3 pathway in CNE2 cells. In Wound healing assay, migration and invasion assays, we found that the effect of down-regulating the invasion ability of CNE2 cells was consistent with silencing CRT expression or inhibiting Smad3 phosphorylation. Moreover, the epithelial marker E-cadherin was up-regulated and the mesenchymal marker Vimentin was down-regulated, indicating that CRT induces NPC cell EMT via Smad3-dependent TGF-β signaling pathway. NRP1 may act as a substrate for the regulation of CRT/Smad3 pathway to induce EMT in NPC cells to promote tumor cell migration and invasion.

On the whole, the current evidences revealed that CRT expression is positively correlated with the occurrence of EMT in NPC cells. NRP1 is an EMT-related protein and a downstream effector molecule of the CRT/Smad3 pathway. Our evidences have confirmed that NRP1 promote the migration and invasion of NPC CNE2. Therefore, we draw a conclusion that the CRT/Smad3 pathway induces EMT in NPC CNE2 cells by regulating the expression of downstream protein NRP1. This will provide a new direction and ideas for the study of CRT-induced EMT migration and invasion mechanism. In addition, it provides a new experimental basis for targeted therapy of NPC. However, we need to further research the way in which CRT activates the Smad3-dependent TGF-β signaling pathway and how Smad3 acts as a transcription factor to regulate the expression of NRP1.

## Supplementary Material

Supplementary table.

## Figures and Tables

**Table 1:** Cell cycle analysis and distribution of apoptotic cells of NP69-pLNSX and NP69-LMP1^WT^.

Cell lines	G1 phase (%)	G2 phase (%)	S phase (%)	Apoptosis rate (%)	Proliferation index (%)
NP69-pLNSX	43	13.9	42.9	2.45	56.8
NP69-LMP1^WT^	24.5	11.2	64.2*	0.84	75.4

NP69-LMP1^WT^ VS NP69-pLNSX, *P <0.05 (χ^2^=6.38).

**Table 2:** Apoptotic cells

Cell lines	Apoptotic cells count
NP69-pLNSX	67.93±2.74
NP69-LMP1^WT^ *	14.13±1.98

NP69-LMP1^WT^ VS NP69-pLNSX, *P<0.05.

**Table 4:** Expression of Calreticulin in NPC and nasopharynx inflammatory

Group	Case	Calreticulin expression rate
nasopharynx inflammatory	57	19.29% (11/57)
NPC	52	82.69% (43/52)*

*Compared with nasopharynx inflammatory tissue, *P*<0.05.

**Table 3:** Identified differential protein spots between NP69-pLNSX and NP69-LMP1^WT^ cell lines

Spot	AC*	Expressed in NP69-LMP1	Expressed in NP69-pLNSX	Ratio NP69-LMP1/ NP69-pLNSX	Protein	pI	Molecular weight (KDa)	Coverage (%)	Function
1	P27797	5.85±0.56	1.33±0.34	4.40↑	Calreticulin	4.29	48.28	35	Calcium binding
2	P08670	1.38±0.45	0.34±0.22	4.03↑	vimentin	5.06	53.65	59	Constitutive protein
3	P07437	1.24±0.32	0.33±0.12	3.76↑	Tubulin beta-2 chain	4.78	50.09	55	Constitutive protein
4	P29692	0.72±0.13	0.2±0.07	3.60↑	Elongation factor 1	4.90	31.21	38	Transcription and translation
5	P02545	2.46±0.87	0.86±0.49	2.86↑	lamin A/C	6.40	65.14	46	Constitutive protein
6	O75390	2.15±0.72	0.92±0.35	2.34↑	citrate synthase	8.45	51.90	45	Metabolic enzymes
7	Q04826	0.85±0.21	0.37±0.17	2.30↑	MHC class I antigen	5.83	31.64	64	Immunoloregulation
8	P10809	6.93±1.12	3.07±0.78	2.26↑	60 kDa heat shock protein	5.70	61.18	40	Molecular chaperone
9	P55084	0.96±0.28	0.46±0.22	2.09↑	Trifunctional enzyme	9.40	51.54	28	Metabolic enzymes
10	P30101	3.37±1.05	7.14±1.34	0.47↓	Protein isulfide-isomerase A3	5.98	57.14	42	Metabolic enzymes
11	P27144	0.27±0.16	0.68±0.14	0.40↓	Adenylate kinase isoenzyme 4	8.47	25.36	51	Metabolic enzymes
12	Q04837	0.82±0.46	2.26±0.82	0.36↓	Single Strand DNA-binding Protein	9.59	17.24	50	Repair of DNA damage
13	P07355	0.51±0.17	1.57±0.32	0.32↓	Annexin A2	7.56	38.67	51	Signal transduction
14	P98179	0.56±0.34	1.84±0.51	0.30↓	RNA binding motif protein 3	8.86	17.16	46	Transcription and translation
15	P61604	0.26±0.14	0.87±0.34	0.30↓	10 kDa heat shock protein	8.91	10.79	33	Molecular chaperone
16	P61604	0.17±0.12	0.58±0.15	0.29↓	chaperonin 10-related protein	8.91	10.79	33	Molecular chaperone
17	P17661	0.34±0.14	1.26±0.48	0.27↓	Desmin	5.21	53.42	31	Molecular chaperone
18	P05783	0.45±0.32	2.46±0.94	0.18↓	cytokeratin 18	5.34	47.89	69	Constitutive protein
19	P05787	0.59±0.44	3.58±1.02	0.16↓	keratin 8	5.52	53.67	50	Constitutive protein
20	P13647	0.28±0.14	2.04±0.64	0.14↓	Keratin, type II cytoskeletal 5	6.90	62.63	37	Constitutive protein
21	P38646	0.08±0.05	0.67±0.12	0.12↓	Stress-70 protein	5.87	73.92	30	Molecular chaperone
22	P08727	0.75±0.62	8.64±2.46	0.09↓	Keratin 19	5.04	44.06	49	Constitutive protein

AC, Swiss-Prot accession number; ↑, spot significantly upregulated in NP69-LMP1^WT^ compared with NP69-pLNSX; ↓, spot significantly downregulated in NP69-LMP1^WT^ compared with NP69-pLNSX. LMP1, latent membrane protein 1; WT, wild type; pI, isoelectric point.

**Table 5:** Calreticulin expression characteristics in nasopharyngeal carcinoma

Characteristic	Cases	Calreticulin positive	R value	P value
Sex				
Men	37	31 (83.78)	0.087	0.450
Female	15	12 (80.00)		
Age (years)				
≥60	14	11(78.57)	0.075	0.574
<60	38	32(84.21)		
Clinical stage				
I	20	12(60.00)	0.457	*0.013
II-III	32	31(96.87)		
Lymph node metastasis				
Yes	33	32(96.96)	0.382	*0.018
No	19	11(57.89)		

Calreticulin expression was positively associated with clinical stage and infiltrated cervical lymph node, *P <0.05.

**Table 6:** Prediction results of the transcription factor SMAD3 in the human NRP1 gene promoter region binding site

Name	Score	Predicted site sequence	From	To	Strand
SMAD3	6.18183	TGACTAGATA	200	209	+
SMAD3	5.45193	TATCTAGTCA	200	209	-
SMAD3	9.51165	AGTCTAGAAA	855	864	+
SMAD3	8.8057	TTTCTAGACT	855	864	-
SMAD3	6.8747	AGCCTAGACC	1109	1118	-

+, Sense strand; -, antisense strand.

## Abbreviations

LMP1: Latent membrane protein 1
EMT: Epithelial-mesenchymal transition
CRT: calreticulin
NPC: nasopharyngeal carcinoma
NRP1: neuropilin-1
TGF-β: transforming growth factor β
EBV: Epstein-Barr virus
aa: amino acid
CTAR: carboxy terminal activating regions
WT: wild type
IHC: Immunohistochemical
