# Supplementary material for: LMP1 Up-regulates Calreticulin to Induce Epithelial-mesenchymal Transition via TGF-β/Smad3/NRP1 Pathway in Nasopharyngeal Carcinoma Cells
Source: J Cancer. 2020 Jan 1;11(5):1257–69. doi: 10.7150/jca.37415 (PMC6959064; doi:10.7150/jca.37415)
Supplement: Supplementary file 1 — Supplementary table. [file jcav11p1257s1.pdf]

Supplementary Table 1

>5' Flanking sequence chromosome:GRCh38:10:33336263:33338262:-1

CTTGCATGACACAGTCACTTGACAGAATAACCACTAATAGGCTTATTTT
AAACATTAGTTAATTAAAATTCCAACTTTTAATGTTTTTCCAGTTTCACTTCA
TCTATTCATACATCTCAAAACTAGGTTAGCTCCTACTAAAAATATGGAGCTT
GTATTGCTTTTCTGAAATTTATGGGTTTGATGTTTTCAAAAAACT**TGACTAG**
ATATGATGCAGTTTGAACTACCAACCCGAATCTCCATATTGCATGAATCTGA
AGTACTTTGAGGGAGTTAGTGGCTCAAATGGCCTTCTGGGAGCAATCGATT
TCATCTATTTCTGACAAAGGGATGAGAGCTTGATGAAAGGAATCGAGCAGT
TACCATCCAGTCTACTCTTACTGATGGGCCACGGTTCCAATCCGACTCAGGGA
GCCCCAGCTGAAGGCCTTATTTCTGCAGCACCTCCTAAGTGGGAGTCCAGGAA
ACTGGGCTTTGCCCACTCACAGCCTCTGCAGGGCGTTCTGGGGGTGCGGTAG
AGGACTCTTCCCTTCCCTGGAACTGGGTCCTACCAAGTCCAGGGATTTGTTCTA
TCAGGAAGAATAATGTGTTCAAGAGACCAAAATAAAATCAACCCGGGAACAC
TGCCAAATTCTTCCTCTGCCTATCACTTATTCTCGTATAGAGCCGGCCTGTCTCC
ACGCTGGGGAATTGCAAAAATCTAAAACTGACCCCTCTCACCACTTAGTGGAA
TCCAACAGCAAAGGAAAAAGGTTGTTAGCAAGGACAATTTGCTCTGTACCAA
CCCACTGGCCGGCAATTCTCAATCAGTGCGGTAGGGCTCCATTACCCAGCACT
GTGAGTGGAAACTAAGG**AGTCTAGAAA**AACCTTAGATCAGCTAAGAGCAGA
GAAATCAGAGGCAGAACTTAAATATGACATTTCCTTCTGAGTCCATAGTGCATC
CCTTATTCACTGGTTGGAGAAAGCTGGCAAAAGCGCTCAGCTGGAGGCAGGC
GTTTTCTGTGCGTTGAGGCGCCGGAGGAGCCATTCCCTTCGGGAAAGACAAG
GTCGGGGTGGGGGCAGTCGCTCCGGAGTGCACGATCCGGTGGCTTTATTTGCT
GGCCTGGCGGGTCTAGGCTGGAAAATGCACACTCAGCAGGGAAAGGGGAAG
AGAGATTGGCGGCAGTTTTCTAAAAACAACACAGCTCAAGTCTCCCTCGTGTG
CCCCCTCGCTCACCCCGCCGAGGCTCAGAGCTATCCAAACGGGCGCTCCGGA
GCGAGCGCGGAGGTCGGGTCCCCCCAGGACGCAGGCGGGCCTCCGAGACTC
CAGGCCGCCCCGGCCGCCGCTCCCTCCCGGTTCCCCTTCCCTGTCAACCTGCC
AGGTCCTGAGAGGCGCCTCTCCCTCCGAGCCTGGACCGAGGTCGCTCCGGGT
CACCCTAAATCTGATGATGCCGCTCGCCCGGCGCAGCCGGGGGCTGGGACTGG
CGGGGCTGCACCTGGGGAGGCGGCGACCGCGCGCGGGTAGTAGCCGAGAGC
CCAGGCTGCCTCTGCGGCGCTCACTCCCCCGGGGTCTGCGCTGGGCGCGGTC
GGCGCCCCCGCCTCGCCTCCCGCCTGCCCGCCTGCCCGGGCGCAGGGCTGCC
CATGGCTTCGACAGTGCTCCTTCCGCTCAACTTAGGCATTTAACTCCCAGCCCT
TCGGTGCCCACTTCCCACCCCGCGCCTCCCAGGGGTCCGACAGCTGAGAAGA
AGGAGGAAACTGGCAGAGGAAAGAGGCGCTCGGGGGGGCCGAGTTTCCAAC
TTGGCGACTGGGAATCCCGAGGCGCGTGGGGAGGAGAAGACAGGCTTGACC

CGGGGCAAGAGAGTGGCGAGTGCGGGCTCCGTGGGTGCACCTCCCACTGGCC
CGGGAACGGCCTGCACCCCGAGGGGCGCACGTCACTGCTCCCAGCCCGAGTC
GCGAGCGCGCAGTCCTGGCCTGGGGCCCGCGCGCCTTGGGCTTTCTCCCTGCC
CGCCCCTTTTTCTTCC
